# Bioceramic-Based Root Canal Sealers: A Review

**DOI:** 10.1155/2016/9753210

**Published:** 2016-05-03

**Authors:** Afaf AL-Haddad, Zeti A. Che Ab Aziz

**Affiliations:** Department of Restorative Dentistry, Faculty of Dentistry, University of Malaya, 50603 Kuala Lumpur, Malaysia

## Abstract

Bioceramic-based root canal sealers are considered to be an advantageous technology in endodontics. The aim of this review was to consider laboratory experiments and clinical studies of these sealers. An extensive search of the endodontic literature was made to identify publications related to bioceramic-based root canal sealers. The outcome of laboratory and clinical studies on the biological and physical properties of bioceramic-based sealers along with comparative studies with other sealers was assessed. Several studies were evaluated covering different properties of bioceramic-based sealers including physical properties, biocompatibility, sealing ability, adhesion, solubility, and antibacterial efficacy. Bioceramic-based sealers were found to be biocompatible and comparable to other commercial sealers. The clinical outcomes associated with the use of bioceramic-based root canal sealers are not established in the literature.

## 1. Introduction

The main functions of root canal sealers are (i) sealing off of voids, patent accessory canals, and multiple foramina, (ii) forming a bond between the core of the filling material and the root canal wall, and (iii) acting as a lubricant while facilitating the placement of the filling core and entombing any remaining bacteria [1]. Due to the relative biological and technical importance of sealers, their chemical and physical properties have been the subject of considerable attention since their initial development in the early twentieth century [2]. Sealers are categorised according to their main chemical constituents: zinc oxide eugenol, calcium hydroxide, glass ionomer, silicone, resin, and bioceramic-based sealers.

Root canal sealers have been reviewed across a number of studies, either collectively [2] or based on their composition, including zinc oxide eugenol [3], calcium hydroxide [4], glass ionomer [5], and resin-based sealers [6]. However, no extensive review of bioceramic-based sealers has been conducted.

Bioceramic-based sealers have only been available for use in endodontics for the past thirty years, their rise to prominence corresponding to the increased use of bioceramic technology in the fields of medicine and dentistry. Bioceramics are ceramic materials designed specifically for medical and dental use. They include alumina, zirconia, bioactive glass, glass ceramics, hydroxyapatite, and calcium phosphates [7]. The classification of bioceramic materials into bioactive or bioinert materials is a function of their interaction with the surrounding living tissue [8]. Bioactive materials, such as glass and calcium phosphate, interact with the surrounding tissue to encourage the growth of more durable tissues [9]. Bioinert materials, such as zirconia and alumina, produce a negligible response from the surrounding tissue, effectively having no biological or physiological effect [8]. Bioactive materials are further classified according to their stability as degradable or nondegradable. Bioceramics are commonly used for orthopaedic treatments, such as joint or tissue replacements, and for coating metal implants to improve biocompatibility. Additionally, porous ceramics, such as calcium phosphate-based materials, have been used as bone graft substitutes [10].

Calcium phosphate was first used as bioceramic restorative dental cement by LeGeros et al. [11]. However, the first documented use of bioceramic materials as a root canal sealer was not until two years later when Krell and Wefel [12] compared the efficacy of experimental calcium phosphate cement with Grossman's sealer in extracted teeth, finding no significant difference between both sealers in terms of apical occlusion, adaptation, dentinal tubule occlusion, adhesion, cohesion, or morphological appearance. Nonetheless, the experimental calcium phosphate sealer failed to provide apical sealing as effectively as Grossman's sealer [13]. Chohayeb et al. [14] later evaluated the use of calcium phosphate as a root canal sealer in adult dog teeth. They reported that the calcium phosphate-based sealer made for a more uniform and tighter adaptation to the dentinal walls as compared to gutta-percha [14]. Calcium phosphate cement has subsequently been used successfully in endodontic treatments, including pulp capping [15], apical barrier formation, periapical defect repairs [16], and bifurcation perforation repairs [17].

There are two major advantages associated with the use of bioceramic materials as root canal sealers. Firstly, their biocompatibility prevents rejection by the surrounding tissues [9]. Secondly, bioceramic materials contain calcium phosphate which enhances the setting properties of bioceramics and results in a chemical composition and crystalline structure similar to tooth and bone apatite materials [18], thereby improving sealer-to-root dentin bonding. However, one major disadvantage of these materials is in the difficulty in removing them from the root canal once they are set for later retreatment or post-space preparation [19].

The exact mechanism of bioceramic-based sealer bonding to root dentin is unknown; however, the following mechanisms have been suggested for calcium silicate-based sealers:

1. Diffusion of the sealer particles into the dentinal tubules (tubular diffusion) to produce mechanical interlocking bonds [20].
2. Infiltration of the sealer's mineral content into the intertubular dentin resulting in the establishment of a mineral infiltration zone produced after denaturing the collagen fibres with a strong alkaline sealer [21, 22].
3. Partial reaction of phosphate with calcium silicate hydrogel and calcium hydroxide, produced through the reaction of calcium silicates in the presence of the dentin's moisture, resulting in the formation of hydroxyapatite along the mineral infiltration zone [23].

While various branded bioceramic-based root canal sealers are available on the market, others are still experimental, requiring further laboratory and clinical testing to ascertain their efficacy. A number of commercially available bioceramic-based root canal sealers, classified according to their major constituents, are identified in Table 1. The biological and physical properties of bioceramic-based root canal sealers were reviewed based on the ideal root canal sealer properties as described by Grossman [24], as in the following list:

1. It should be tacky when mixed to provide good adhesion between it and the canal wall when set.
2. It should make a hermetic seal.
3. It should be radiopaque so that it can be visualized on the radiograph.
4. The particles of powder should be very fine so that they can mix easily with liquid.
5. It should not shrink upon setting.
6. It should not discolour tooth structure.
7. It should be bacteriostatic or at least not encourage bacterial growth.
8. It should set slowly.
9. It should be insoluble in tissue fluids.
10. It should be well tolerated by the periapical tissue.
11. It should be soluble in common solvents if it is necessary to remove the root canal filling.

## 2. Ideal Root Canal Sealer Properties

### 2.1. Biocompatibility

Biocompatibility is an essential requirement of any root canal sealer as the root filling material constitutes a true implant coming into direct contact with the vital tissue at the apical and lateral foramina of the root or indirectly via surface restoration [2]. Biocompatibility is defined as the ability of a material to achieve a proper and advantageous host response in specific applications [25]. In other words, a material is said to be biocompatible when the material coming into contact with the tissue fails to trigger an adverse reaction, such as toxicity, irritation, inflammation, allergy, or carcinogenicity [26]. Most studies assess biocompatibility through investigations of cytotoxicity, in reference to the effect of the material on cell survival [27]. The cytotoxicity of bioceramic-based sealers has been evaluated* in vitro *using mouse and human osteoblast cells [28, 29] and human periodontal ligaments cells [30]. Most bioceramic-based root canal sealers have subsequently been found to be biocompatible. This biocompatibility is attributed to the presence of calcium phosphate in the sealer itself. Calcium phosphate also happens to be the main inorganic component of the hard tissues (teeth and bone). Consequently, the literature notes that many bioceramic sealers have the potential to promote bone regeneration when unintentionally extruded through the apical foramen during root canal filling or repairs of root perforations [30, 31].

Sankin apatite has been shown by Telli et al. [32] to be biocompatible in* in vitro *studies. However, Kim et al. [33] showed that Sankin apatite exerts a tissue response when implanted subcutaneously in rats and that this response began to subside within two weeks. The biocompatibility of Sankin apatite root canal sealer was also evaluated in comparison to an experimental calcium phosphate-based sealer composed of tetracalcium phosphate, dicalcium phosphate dihydrate, and modified McIlvaine's buffer solution. Yoshikawa et al. [34] found that Sankin apatite caused severe inflammatory reactions in both the dorsal subcutaneous and the periapical tissue of rats. However, the experimental sealer produced no inflammatory response in the subcutaneous tissue and only a mild reaction in the periapical tissue [34]. The cytotoxicity of the Sankin apatite root canal sealer is the result of the presence of iodoform and polyacrylic acids in the sealer [33]. However, Sankin apatite type II and type III were found to be more biocompatible than either type I or Grossman's sealer [35]. EndoSequence BC, iRoot SP, and MTA-Fillapex showed moderate toxicity when freshly mixed; however, cytotoxicity reduced over time until being completely set [29, 36, 37]. Although* in vitro* evaluations of biocompatibility can be an indicator of the cytotoxicity of a material,* in vitro *immunological deficiencies should be taken into consideration. Some sealers have been shown to have severe cytotoxicity* in vitro*, such as zinc oxide eugenol-based sealers; however, such toxicity is not necessarily clinically significant [38].

Capseal I and Capseal II sealers have been shown to produce less tissue irritation and less inflammation compared to other sealers [30, 31, 33]. Shon et al. [39] studied the effects of Capseal I and Capseal II in comparison to Sankin apatite root sealer (type I and type III) and a zinc oxide eugenol-based sealer (Pulp Canal Sealer). Investigators exposed human periodontal fibroblast cells to the various sealers before measuring the inflammatory response by way of inflammatory mediators and the viability and osteogenic potential of osteoblast MG63 cells. They found Capseal I and Capseal II to possess low cytotoxicity and to facilitate periapical dentoalveolar healing by regulating cellular mediators from periodontal ligaments cells and osteoblast differentiation. MTA-Fillapex was found to have a severe cytotoxic effect on fibroblast cells when freshly mixed. Furthermore, this effect did not decrease with time. The level of cytotoxicity remained moderate even five weeks after mixing [40].

### 2.2. Setting Time

The ideal root canal sealer setting time should permit adequate working time. However, a slow setting time can result in tissue irritation, with most root canal sealers producing some degree of toxicity until being completely set. According to the manufacturers of EndoSequence BC Sealer or iRoot SP, the setting reaction is catalysed by the presence of moisture in the dentinal tubules. While the normal setting time is four hours, in patients with particularly dry canals, the setting time might be considerably longer [41]. The amount of moisture present in the dentinal tubules of the canal walls can be affected by absorption with paper points [42], the presence of smear plugs, or tubular sclerosis [43].

Loushine et al. [28] reported that EndoSequence BC Sealer requires at least 168 hours before being completely set under different humidity conditions, as evaluated using the Gilmore needle method. Zhou et al. [44], on the other hand, reported a setting time of 2.7 hours. The setting reaction of EndoSequence BC Sealer is a two-phase reaction. In phase I, monobasic calcium phosphate reacts with calcium hydroxide in the presence of water to produce water and hydroxyapatite. In phase II, the water derived from the dentin humidity, as well as that produced by the phase I reaction, contributes to the hydration of calcium silicate particles to trigger a calcium silicate hydrate phase [28].

The manufacturer of MTA-Fillapex claims that their product will set in a minimum of two hours and this setting time has been confirmed in at least two studies [44, 51]. However, even shorter setting times for MTA-Fillapex (66 min) have been reported [52]. The setting reaction of MTA material is complicated and has been discussed by Darvell and Wu [53]; however, the setting reaction of MTA-based sealers has not been described in the literature.

### 2.3. Flow

Flow is an essential property that allows the sealer to fill difficult-to-access areas, such as the narrow irregularities of the dentin, isthmus, accessory canals, and voids between the master and accessory cones [54]. According to ISO 6786/2001 [55], a root canal sealer should have a flow rate of not less than 20 mm. Factors that influence the flow rate of the sealer include particle size, temperature, shear rate, and time from mixing [4]. The internal diameter of the tubes and rate of insertion are considered when assessing flow rate via the Rheometer method [2]. The flow rate for EndoSequence BC Sealer has been variously reported as 23.1 mm and 26.96 mm [44, 54]. Similarly, the flow rate of MTA-Fillapex has been variously reported as 22 mm, 24.9 mm, and 29.04 mm [44, 51, 52]. While most of the bioceramic-based root sealer manufacturers included in Table 1 claim that the flow rate of their sealers meets ISO requirements, the literature does not support such claims.

### 2.4. Retreatability

Root filling materials provide a mechanical barrier for the isolation of necrotic tissue or bacteria responsible for the persistence of periapical inflammation or postoperative pain [56, 57]. Wilcox et al. [58] observe that most of the remaining material during retreatment is sealer. Therefore, the complete removal of the sealer is essential during endodontic retreatment to establish healthy periapical tissues. EndoSequence BC Sealer is difficult to remove from the root canal using conventional retreatment techniques, including heat, chloroform, rotary instruments, and hand files. A number of cases have been reported in which obstruction of the apical foramen has resulted in a loss of patency [59]. By contrast, Ersev et al. [60] reported that the removability of EndoSequence BC Sealer from the root canal is comparable to AH Plus. Sankin apatite root canal sealer is easily removed during retreatment with and without the use of solvents [61]. Retreatability with MTA-Fillapex is comparable to that of AH Plus in terms of material remaining in the canal, dentin removal, and time taken to reach working length [62].

### 2.5. Solubility

Solubility is the mass loss of a material during a period of immersion in water. According to ANSI/ADA Specification 57 [63], the solubility of a root canal sealer should not exceed 3% by mass. A highly soluble root canal sealer would invariably permit the formation of gaps within and between the material and the root dentin, thereby providing avenues for leakage from the oral cavity and periapical tissues [2].

Both iRoot SP and MTA-Fillapex are highly soluble, 20.64% and 14.89%, respectively, which does not meet ANSI/ADA requirements [52, 64]. This high solubility is the result of hydrophilic nanosized particles being present in both sealers which increases their surface area and allows more liquid molecules to come into contact with the sealer. However, the literature contains conflicting accounts, with Viapiana et al. [52] finding MTA-Fillapex to be highly soluble and Vitti et al. [51] reporting the solubility of MTA-Fillapex to be <3%, consistent with ISO 6876/2001. Similarly, the solubility of EndoSequence BC is reported to be consistent with ISO 6876/2001 [44]. This discrepancy between the findings of these studies might be attributed to variations in the methods used to dry the samples after having subjected them to solubility testing. The low solubility of MTA-Angelus, consistent with ANSI/ADA requirements [64], is the result of an insoluble matrix of crystalline silica present within the sealer that maintains its integrity even in the presence of water [65].

### 2.6. Discolouration of Tooth Structure

For reasons of aesthetic appearance, a root canal sealer should not stain the tooth. The chromogenic effects of root sealers are increased when excess sealer is not removed from the coronal dentin of the pulp chamber [66]. Partovi et al. [67] observe that Sankin apatite III results in the least discolouration nine months after application as compared with AH26, Endofill, Tubli-Seal, and zinc oxide eugenol sealers. The greatest degree of discolouration was observed following treatment of the cervical third of the crown [67]. MTA-Fillapex was found to cause the least crown discolouration to the extent of not being clinically perceptible [66].

### 2.7. Radiopacity

Root canal sealers should be sufficiently radiopaque so as to be distinguishable from adjacent anatomical structures [68]. This allows the quality of the root filling to be evaluated through radiographic examination. According to ISO 6876/2001, the minimum radiopacity for a root canal sealer is based on a reference standard of 3.00 mm of aluminium. Candeiro et al. [54] reported the radiopacity of EndoSequence BC Sealer to be 3.83 mm. Endo CPM sealer was found to have a radiopacity of 6 mm due to the presence of bismuth trioxide and barium sulphate [69]. Similarly, the presence of bismuth trioxide in MTA-Fillapex gives it a radiopacity of 7 mm [52, 70].

### 2.8. Antimicrobial Properties

The antimicrobial activity of a root canal sealer increases the success rate of endodontic treatments by eliminating residual intraradicular infections that might have survived root canal treatment or have invaded the canal later through microleakage [71, 72]. According to the literature, the key antimicrobial properties of root canal sealers lie in their alkalinity and release of calcium ions [4] which stimulates repair via the deposition of mineralised tissue [73].

Two methods are commonly used to evaluate the antibacterial activity of bioceramic-based root canal sealers: the agar diffusion test [74, 75] and direct contact testing [23, 75]. EndoSequence BC Sealer has been shown to have high pH (>11) as well as high tendency to release calcium ions [54]. Zhang et al. [23] tested the antibacterial activity of iRoot SP sealer* in vitro *against* Enterococcus faecalis* through a modified direct contact test, finding that iRoot SP sealer had a high pH value (11.5) even after setting but that its antibacterial effect was greatly diminished after seven days. The investigators suggested two additional mechanisms associated with the antibacterial efficacy of iRoot SP: hydrophilicity and active calcium hydroxide diffusion [23]. Hydrophilicity reduces the contact angle of the sealer and facilitates penetration of the sealer into the fine areas of the root canal system to enhance the antibacterial effectiveness of iRoot SP* in vivo *[23].

Morgental et al. [75] evaluated the antibacterial activity of MTA-Fillapex and Endo CPM against* Enterococcus faecalis* using an agar diffusion test after mixing and a direct contact test after setting. The pH of the Endo CPM suspension was greater than that of MTA-Fillapex (>11); however, the bacterial inhibition zone produced by MTA-Fillapex was greater than that produced by Endo CPM [75]. The investigators attributed the antibacterial activity of MTA-Fillapex to the presence of resin as a core ingredient. Nevertheless, neither sealer was able to sustain its antibacterial activity after setting despite their initial high pH levels [75].

*Enterococcus faecalis* is the most common intraradicular microbe isolated from periapical periodontitis [76, 77] and is therefore commonly used to test the antibacterial activity of root canal sealers. Other microorganisms, such as* Micrococcus luteus*,* Staphylococcus aureus*,* Escherichia coli*,* Pseudomonas aeruginosa*,* Candida albicans*, and* Streptococcus mutans*, have also been used to test the antibacterial effects of bioceramic-based sealers [74, 78]. Freshly mixed Endo CPM exhibits antibacterial activity against* Staphylococcus aureus* and* Streptococcus mutans* with no significant reduction of the inhibition zone after setting. Nevertheless, the antibacterial effect is less than that of AH-26 [78]. MTA-Angelus has an antibacterial effect against* Micrococcus luteus*,* Staphylococcus aureus*,* Escherichia coli*,* Pseudomonas aeruginosa*, and* Candida albicans *[74].

### 2.9. Adhesion

Root canal sealer adhesion is defined as its capacity to adhere to the root canal dentin and promote GP cone adhesion to each other and the dentin [79]. Tagger et al. [80] argued that the term* adhesion* should be replaced with* bonding* in the case of root canal sealers because the attachment between the substances involves mechanical interlocking forces rather than molecular attraction. There is no standard method used to measure the adhesion of a sealer to the root dentin; therefore, the adhesion potential of the root filling material is commonly tested using microleakage and bond strength tests [81].

The sealing ability of a sealer is related to its solubility and to its bonding to the dentin and root canal filling cones [4]. Several studies have evaluated the sealing abilities of different bioceramic-based sealers* in vitro*. These studies are summarised in Table 2. Regardless of the different methodologies used, the sealing ability of bioceramic-based sealers has been found to be satisfactory and comparable to other commercially available sealers. However, until recently, there had been a paucity of literature concerning the long-term sealing ability or clinical outcomes associated with bioceramic-based sealers.

Bond strength is the force per unit area required to debond the adhesive material from the dentin [81]. Although no correlation has been identified between leakage and bond strength [82], the bond strength test has received significant attention due to the development of the “monoblock” concept in which a sealer bonds to both the core material and the dentinal wall to create a singular unit that enhances sealing and strengthens the root-filled tooth against fracture [83]. A strong bond between the root canal sealer and the root dentin is essential for maintaining the integrity of the sealer-dentin interface during the preparation of post-spaces and during tooth flexure [84]. Bioceramic-based sealers have the ability to create bonds between the dentin and core filling materials [9]. The bonding of iRoot SP to root dentin is comparable to that of AH Plus and stronger than either Sealapex or EndoREZ sealers [85]. Shokouhinejad et al. [86] evaluated the bond strength of EndoSequence BC Sealer compared to AH Plus in the presence and absence of a smear layer, finding that the dislocation resistance of EndoSequence BC Sealer was equal to that of AH Plus and with no significant effect on the smear layer. Nagas et al. [87] studied the bond strengths of several sealers under various moisture conditions present in the root canal, concluding that a sealer's bond strength is greatest in moist and wet canals, the presence of residual moisture positively affecting the adhesion of the root canal sealers to radicular dentin. As compared with AH Plus, Epiphany, and MTA-Fillapex, iRoot SP had the highest dislodgment resistance from the root dentin [87]. Moreover, the prior placement of intracanal calcium hydroxide improved the bonding of iRoot SP to the root dentin; however, the bonding was less than that of AH Plus and comparable to MTA-Fillapex in the absence of calcium hydroxide [88]. This improvement in bonding is explained by way of the chemical interaction between calcium hydroxide and the iRoot SP sealer increasing the frictional resistance and/or micromechanical retention of the sealer [88]. Endo CPM has a significantly higher bond strength compared to MTA-Fillapex or AH Plus [89].

Testing the bond strength at the coronal third of the root canal shows no significant difference between MTA-Fillapex, iRoot SP, and AH Plus. However, in middle and apical thirds, iRoot SP and AH Plus have equivalent bond strengths superior to MTA-Fillapex [90]. Huffman et al. [84] tested the dislocation resistance of ProRoot Endo Sealer, AH Plus Jet, and Pulp Canal Sealer from root dentin with and without immersion in a simulated body fluid (SBF). The investigators concluded that ProRoot Endo Sealer possesses greater bond strength than the other two sealers, especially after SBF immersion. According to Huffman et al. [84], the greater bonding of the ProRoot Endo Sealer is due to the presence of spherical amorphous calcium phosphate and apatite-like phases enhancing frictional resistance. There was no negative effect of the iRoot SP root canal sealer on the push-out bond strength of fibre posts cemented with self-adhesive resin cement [91]. Compared to Activ GP sealer (glass ionomer-based sealer, Brasseler USA, Savanah, GA), iRoot SP was found to increase the fracture resistance of endodontically treated roots* in vitro*, a potential indicator of the high bond strength of the sealer [92].

## 3. Conclusion

Bioceramic-based root canal sealers show promising results as root canal sealers. However, discrepancies in the results of these studies reveal that these sealers do not fulfil all of the requirements demanded of the ideal root sealer. The biocompatibility and biomineralization effect of these sealers might avail them for alternative uses in direct pulp capping and root end filling. Further studies are required to clarify the clinical outcomes associated with the use of these sealers.

## Figures and Tables

**Table 1:** Examples of bioceramic-based root canal sealers.

Type	Brand name	Manufacturer	Components
Calcium silicate-based sealer	iRoot SP	Innovative BioCeramix Inc., Vancouver, Canada	Zirconium oxide, calcium silicates, calcium phosphate, calcium hydroxide, filler, and thickening agents
	EndoSequence BC Sealer	Brasseler USA, Savannah, GA, USA	

MTA-based sealer	MTA-Fillapex	Angelus, Londrina, PR, Brazil	Salicylate resin, diluting resin, natural resin, bismuth trioxide, nanoparticulate silica, MTA, and pigments
	Endo CPM sealer	Egeo, Buenos Aires, Argentina	Silicon dioxide, calcium carbonate, bismuth trioxide, barium sulfate, propylene glycol alginate, sodium citrate, calcium chloride, and active ingredients
	MTA-Angelus	Angelus, Londrina, PR, Brazil	Tricalcium silicate, dicalcium silicate, tricalcium aluminate, tetracalcium aluminoferrite, bismuth oxide, iron oxide, calcium carbonate, magnesium oxide, crystalline silica, and residues (calcium oxide, free magnesium oxide, and potassium and sodium sulphate compounds)
	ProRoot Endo Sealer	DENTSPLY Tulsa Dental Specialties	Powder: tricalcium silicate, dicalcium silicate, calcium sulphate, bismuth oxide, and a small amount of tricalcium aluminate Liquid: viscous aqueous solution of a water-soluble polymer

Calcium phosphate-based sealer	Sankin apatite root canal sealer (I, II, and III)	Sankin Kogyo, Tokyo, Japan	Powder: alpha-tricalcium phosphate and hydroxy-Sankin apatite in type I, iodoform added to powder in type II (30%) and type III (5%) Liquid: polyacrylic acid and water
	Capseal (I and II)	Experimental [45]	Powder: tetracalcium phosphate (TTCP) and dicalcium phosphate anhydrous (DCPA), Portland cement (gray cement in type I and white cement in type II), zirconium oxide, and others as powder liquid: hydroxypropyl methyl cellulose in sodium phosphate solution

**Table 2:** Sealing ability of bioceramic-based root canal sealers.

Tested sealers	Compared sealers	Obturation technique	Microleakage site	Microleakage test	Duration	Finding	References
Capseal I and Capseal II	Sankin apatite, AH Plus, Sealapex, and zinc oxide-based sealer (Pulp Canal Sealer-Kerr)	Lateral condensation technique	Apical	Anaerobic bacterial leakage	Every day for a period of 90 days	Capseal I and Capseal II especially Capseal II showed good sealing ability, comparable to that of AH Plus	Yang et al. [45]

Endo CPM and Experimental MTA-based sealer	AH Plus, Sealer 26, Epiphany SE, Sealapex, Activ GP, and Endofill	Cold lateral condensation	Coronal	Bacterial leakage	Every 24 h for a period of 120 days	Activ GP, Endo CPM sealer, and MTAS were less resistant to leakage	Oliveira et al. [46]

Experimental calcium phosphate injectable sealer	Sealapex	Lateral condensation for Sealapex group and single silver cone for experimental sealer	Apical	Poly-R dye penetration test	5 days	Sealing ability was satisfactory in both groups with no significant difference	Cherng et al. [19]

Experimental MTA and fluorodoped MTA (FMTA) sealers	AH Plus	Warm vertical compaction	Apical	Fluid filtration method	24 hours, 48 hours, 1 week, 2 weeks, 1 month, 3 months, and 6 months	FMTA and AH Plus had a significantly better sealing ability than MTA	Gandolfi and Prati [47]

iRoot SP	AH Plus	Continuous wave condensation technique with both sealers and single cone technique with iRoot SP	Apical	Fluid filtration method	24 hours, 1 week, 4 weeks, and 8 weeks	iRoot SP was equivalent to AH Plus sealer in apical sealing ability	Zhang et al. [20]

MTAS	Pulp Canal Sealer	Warm vertical compaction technique	Coronal	Fluid filtration method	1 day and 28 days	MTAS had sealing ability comparable to Pulp Canal Sealer	Camilleri et al. [48]

ProRoot Endo Sealer (DENTSPLY Tulsa Dental Specialties)	Pulp Canal Sealer and AH Plus	Warm vertical compaction technique	Coronal	Fluid filtration method	7 days and 35 days	Sealing ability of ProRoot Endo Sealer and AH Plus is better than that of Pulp Canal Sealer	Weller et al. [49]

Sankin apatite root sealer types I, II, and III	Roth's sealer, Sealapex, and Kerr root canal sealer	Lateral-vertical condensation technique	Apical	Dye penetration using silver nitrate	Not stated	Sealing ability of Sankin apatite II was second better after Sealapex	Barkhordar et al. [50]

Sankin apatite types I, II, and III	Grossman's sealer	Lateral condensation technique	Apical	Methylene blue dye	48 hours	All sealers showed minimal leakage with no significant difference	Bilginer et al. [35]
